# Desulfurization of liquid fuels using aluminum modified mesoporous adsorbent: towards experimental and kinetic investigations

**DOI:** 10.1038/s41598-021-88439-6

**Published:** 2021-04-23

**Authors:** Mohammad Reza Khosravi-Nikou, Mohammad Hadi Safari, Amir Asadi Rad, Pouya Hassani, Mohammad Mohammadian, Maryam Ahmadi, Negin Ghafari, Maryam Naseri

**Affiliations:** 1 Department of Gas Engineering, Ahwaz Faculty of Petroleum, Petroleum University of Technology, Ahwaz, Iran; 2 Department of Chemical Engineering, Abadan Faculty of Petroleum, Petroleum University of Technology, Abadan, Iran; 3 Department of Chemical Engineering, Faculty of Engineering, Golestan University, Aliabad Katoul, Iran

**Keywords:** Chemistry, Engineering, Materials science, Nanoscience and technology

## Abstract

In this study, a modified mesoporous adsorbent (MSU-S) impregnated by aluminum was used to remove the aromatic sulfur compounds from n-decane as the model fuel. Physical and chemical properties of as-synthesized adsorbent were investigated by XRD (X-Ray Diffraction), SEM (Scanning Electron Microscopy), FTIR (Fourier Transform Infrared spectroscopy) and BET (Brunauer–Emmett–Teller) method. Adsorptive desulfurization of model fuel was studied through batch and continues processes under mild temperature and normal atmospheric pressure. The equilibrium adsorption was modeled by Langmuir, Temkin, and Freundlich and the kinetics of adsorption was studied through first, second and intraparticle diffusion models. It was figured out that Temkin and the pseudo-second-order model were best fitting the adsorption equilibrium and describing the kinetics, respectively.

## Introduction

Sulfur combinations present in fuel lead to sulfur dioxide and sulfate particulate generation and they are a major source of environmental pollutions^1,2^. When reduction of CO and NO_x_ are intended in the automobile catalytic guards, organic sulfur compounds can reduce efficiently by poisoning of the catalysts^3,4^. Therefore, fuel desulfurization is one of the most important research areas to overcome this negative effect.

The main sulfur containing compounds (SCCs) in the liquid fossil fuels are thiophene (T) and its derived compounds such as, benzothiophene (BT), dibenzothiophene (DBT) and 4,6- dimethyldibenzothiophene (4,6-DMDBT). There are also some other low molecular weight compounds such as SO_2_, SO_3_, H_2_S which are produced during the combustion and degradation process^5,6^.

To reach the acceptable level of SCCs in the fuel, petroleum refineries use hydrodesulfurization technology (HDS) to reduce sulfur content of hydrocarbon products. But this technic is not ecconomic and effective for sulfur compounds removal because it needs high temperature and pressure and therefore, high operational costs^1–3,7–10^. In order to overcome these difficulties for production of the ultra-clean fuel, alternative methods are investigated. Among of these emerging technologies, adsorptive desulfurization is an alternative to the HDS due to its mild operating conditions. In this process, the removal efficiency can be varied by the physico-chemical properties of adsorbents^11^. For this purpose, different classes of the adsorbents such as reduced metals^12^, metal oxides^13^, metal organic frameworks^14^, zeolite-based materials^15^, carbon or carbon-based composite materials are investigated. Until now, zeolite-based compounds named MSU-S are considered significantly because of their available wide sources, low costs, high surface area and ease of modification in structure^16–19^.

One of the main disadvantages of MSU-S mesoporous materials is the low selectivity towards SCCs. In other words, MSU-S adsorption capability of SCCs and other competitive components in the fuel is almost the same. Therefore, developing highly selective MSU-S with enough loading capacity is important to enhance the adsorption of sulfur compounds in the fuel. In the previous work, different modifications like CeO_2_-MSU-S, Cu_2_O-MSU-S, CoO_2_-MSU-S, Fe_2_O_3_-MSU-S and CrO_2_-MSU-S are introduced and it was obtained that modification by copper has the best performance among other metals to remove sulfur containing compounds^3,20,21^.

The main goal of this work was to remove sulfur components in the synthetized fuel using Al_2_O_3_-MSU-S. The properties of as-synthesized sorbent was examined by BET, XRD, FESEM and FT-IR techniques. Batch and continuous adsorption tests were performed and the adsorption was investigated thermodynamically and kinetically.

## Experimental

### Chemicals

Sodium aluminate and fumed silica were prepared from Sigma-Aldrich Chemical Co. (USA). N-decane, tetrapropylammonium hydroxide (TPAOH 40% aqueous solution), dibenzothiophene, hexa decyl trimethylammonium bromide (HTABr) of synthetic grade, ethanol (> 99%), nonahydrated aluminum nitrate of analytical grade were bought from Merck Co. These compounds were employed without any purifying process.

### Preparation of material

#### MSU-S

The methodology used to synthesize MSU-S (MFI) mesoporous adsorbent was like what is explained by Rashidi et al.^21^. 79.26 g of deionized water was blended with 10.2 g of TPAOH for MFI formation resource. After that sodium aluminate (0.34 g) and fumed silica (6.0 g) were blended in the TPAOH. Then it was blended in an Erlenmeyer flask (braced completely to inhibit water vaporization while aging and to hold the chemicals composition ratio constant) for 18 h at the water bath of 50° C to obtaining zeolite MFI grains. So, HTABr (9.44 g) as the surfactant was blended with deionized water (100 g) and added to the suspension of grains to make the mesoporous structure. Afterward, sulfuric acid (0.1 M) was injected to the chemicals to retain pH at 9. The produced gel was placed in the stainless-steel autoclave, then kept in the oven at 150 °C for 48 h. The produced powder was washed several times using DW and then filtered and placed at 80 °C oven for 9 h. After that, the white chemical was introduced to the mixture of 0.1 M NH_4_NO_3_ and ethanol with a reflux temperature for ion exchanging. The resulting material was desiccated at 90 °C for 12 h. The final product was calcinated in the muffle furnace at 550 °C in the air at 1 °C/min for 10 h.

#### Impregnation

Al (NO_3_)_3_ used as an aluminum source to be loaded on MSU-S. In order to make a dispersed phase of Al_2_O_3_ on the MSU-S, the required amount of metal precursor was mixed in DW. A specific amount of MSU-S was introduced to the mixture while it was mixed and left over the night at 70 °C to lose moisture. The prepared gel was desiccated at 90 °C for 12 h. After that, for the calcination process, the final product was put in the furnace and heated with the rate of 1 °C/min to reach 550 °C and preserved at this temperature for 4 h. In order to activate the synthesized material and before being used in the adsorption test, it was placed at hydrogen atmosphere at 190 °C.

### Model fuel

To prepare synthesized fuel like a real fuel, n-decane as the paraffinic compound and dibenzothiophene as the sulfur source were used.

### Characterization of samples

The XRD (X-ray diffraction) was studied applying wave length = 1.5418 Å, 40 kV and 40 mA of Cu Kα radiation in the domain from 1° to 10° and 20° to 80°. The Fourier transform infrared (FTIR) pattern of the adsorbent was reported on the Nicolet Impact 400. 128 scans were picked at a 4 cm^−1^ resolution in the mid-IR region using KBr. The Brunauer–Emmett–Teller (BET) was applied to derive surface area and average pore diameter. The textural properties of the materials were investigated by N_2_ adsorption/desorption at − 196 °C with the Belsorp-mini-instrument. SEM (scanning electron microscopy) was done on the Zeiss instrument for the morphological study of the sorbent. The chemical analysis of Energy dispersive X-ray spectroscopy (EDS) was performed with Oxford Instrument.

### General adsorption tests

#### Batch process

Batch experiment is done to discuss equilibrium and kinetics of adsorption of DBT on Al_2_O_3_-MSU-S and then to investigate adsorption capacity, equilibrium and kinetics models. For all experiments, 0.2 g adsorbent and 0.5 g model fuel were used. The tests were performed in the capped vials and mixed with constant stirring speed of 250 rpm at room temperature and atmospheric conditions.

#### Continuous adsorption experimental procedure

In order to get the breakthrough and saturation amount for the adsorbent, the experiments were performed in a continuous process. For the continuous tests, DBT content in the fuel was considered to be 400 ppmw. The breakthrough /adsorption experiments were done in the reactor of fixed-bed stainless-steel (150 mm long, 10 mm internal diameter) with a layer of quartz sand. Approximately, 0.5 g of Al_2_O_3_-MSU-S was placed into the reactor. In order to obtain the saturation point the treated fuel was sampled every 5–10 min.

### Sulfur concentration analysis

The sulfur amount in the synthesized fuel was defined by GC Gas Chromatograph YL6100 model with the capillary column (5 TRB, Length: 30 m, Internal Diameter: 0.32 mm, DF: 1 μm; Teknokroma.), and the FID containing He as carrier gas.

## Results and discussion

### Adsorbent features

#### XRD

**Figure 1:**
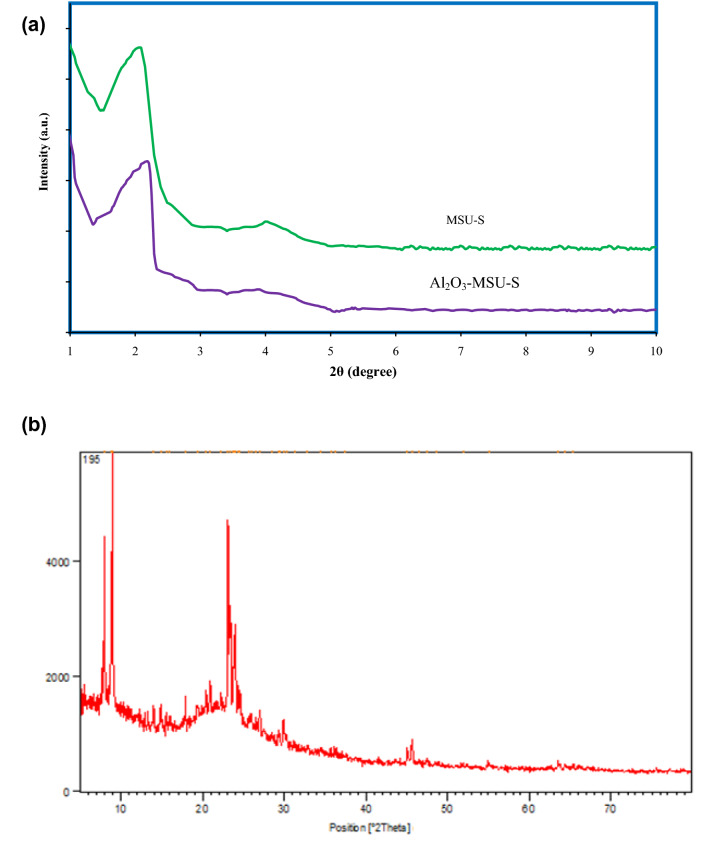
(**a**) XRD with Small-angle pattern for MSU-S and Al_2_O_3_-MSU-S (**b**) XRD with wide-angle pattern of Al_2_O_3_-MSU-S.

**Figure 2:**
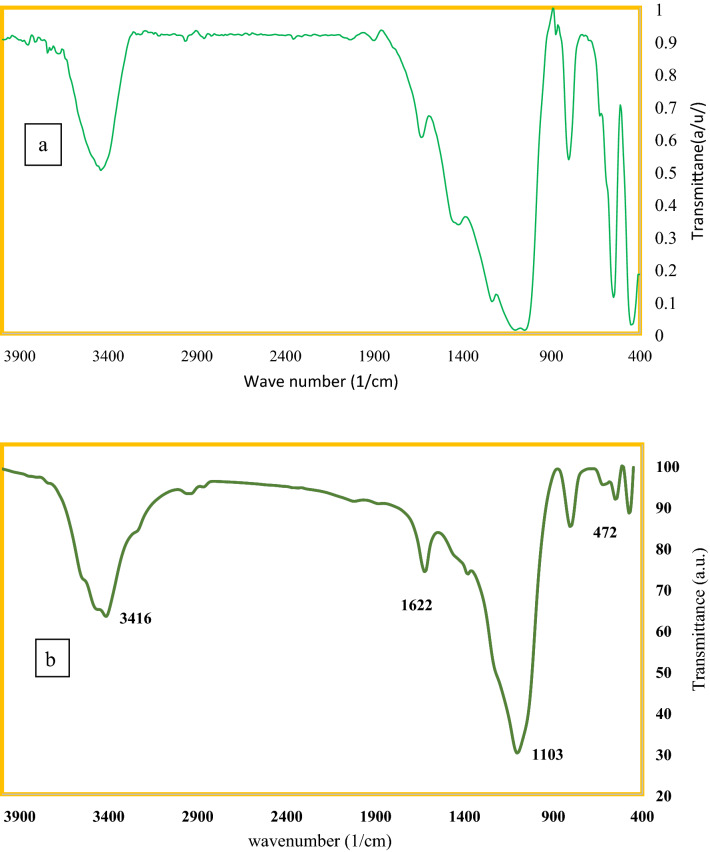
FTIR spectra of two adsorbents. (**a**) MSU-S and (**b**) Al_2_O_3_-MSU-S.

Figure 1a shows the XRD low-angle spectra of the adsorbents. As it illustrate, the influence of loading aluminum decreases maximum intensity of MSU-S clearly, which depicts that crystalline phase converts to amorphous phase relatively^24–26^. It is obvious that impregnating of aluminum has a negative impact on mesoporous pattern of MSU-S. As illustrated in Fig. 1b, there are four maximums in the high-angle area of XRD spectra of Al_2_O_3_-MSU-S, associated with the head for MSU-S hexagonal mesostructure. All these maximum points indicate the formation of crystalline phase of aluminum oxide on the adsorbent (MSU-S) after being injected on the surface^6^ (Fig. 2).

#### Nitrogen adsorption–desorption

**Figure 3:**
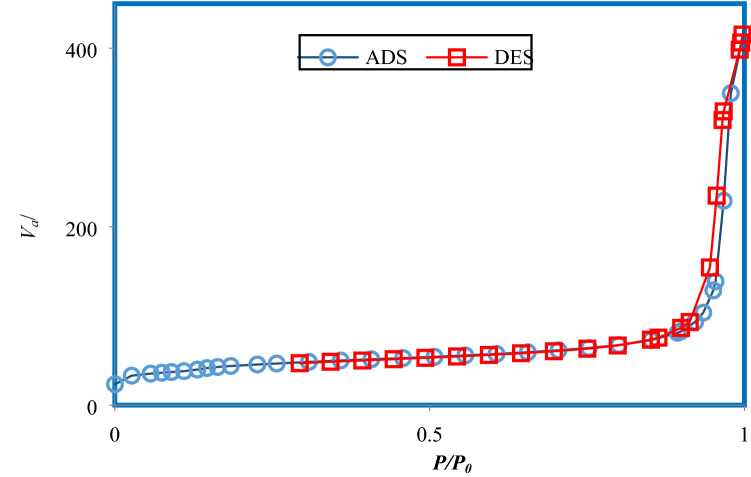
Nitrogen adsorption–desorption isotherm for Al_2_O_3_-MSU-S.

**Figure 4:**
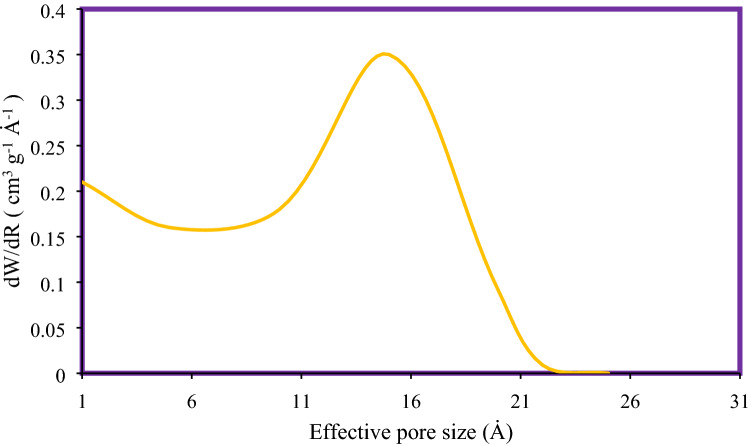
Pore size distribution for Al_2_O_3_-MSU-S.

Volume, mean diameter of the pores and BET surface area for MSU-S and Al_2_O_3_-MSU-S are presented in Table 1. It shows that the surface area of MSU-S was decreased when impregnated by aluminium. Nitrogen adsorption–desorption isotherm is demonstrated in Fig. 3. It is obvious that there is a hysteresis loop during adsorption–desorption. According to the IUPAC classification, isotherm type IV can be used to describe hysteresis behavior of Al_2_O_3_-MSU-S that is the characteristic feature of mesoporous solid. A low-pressure hysteresis which is gained in Fig. 3 extending to the lowest achievable pressures. A step occurs in the adsorption curve between partial pressures P/Po of 0.8 to 1. Figure 4 illustrates the pore size dispensation of Al_2_O_3_-MSU-S. The sharp peak around 21 Å in this figure might be according to the internal cavity and represents the narrow pore size^28–33^.

**Table 1:** Textural features of the sorbents.

Sorbent	BET surface area (m^2^/g)	Mean pore diameter (Å)	Total pore volume (cm^3^/g)
MSU-S	677	24	0.65
Al_2_O_3_-MSU-S	158	21	0.60

#### Fourier transform infrared (FTIR) pattern

The FTIR pattern of MSU-S and Al_2_O_3_-MSU-S is presented in Fig. 2. It indicates that Si–O–Si bond is asymmetric internally and stretches at 1103 per cm. The Si–O–Si proportioned length exists at 472 per cm is related to the Si–O–Al in the adsorbent. The band at 3416 per cm related to the silanol group that is the characteristic of adsorbent. The different band that appears in this adsorbent in comparison with spectrum of MSU-S, centered at 1622 cm^−1^. So, it is concluded that nitrate of the metal reacted with the empty places Al2O3-MSU-S^20,27,28^.

#### FESEM image and EDS result

**Figure 5:**
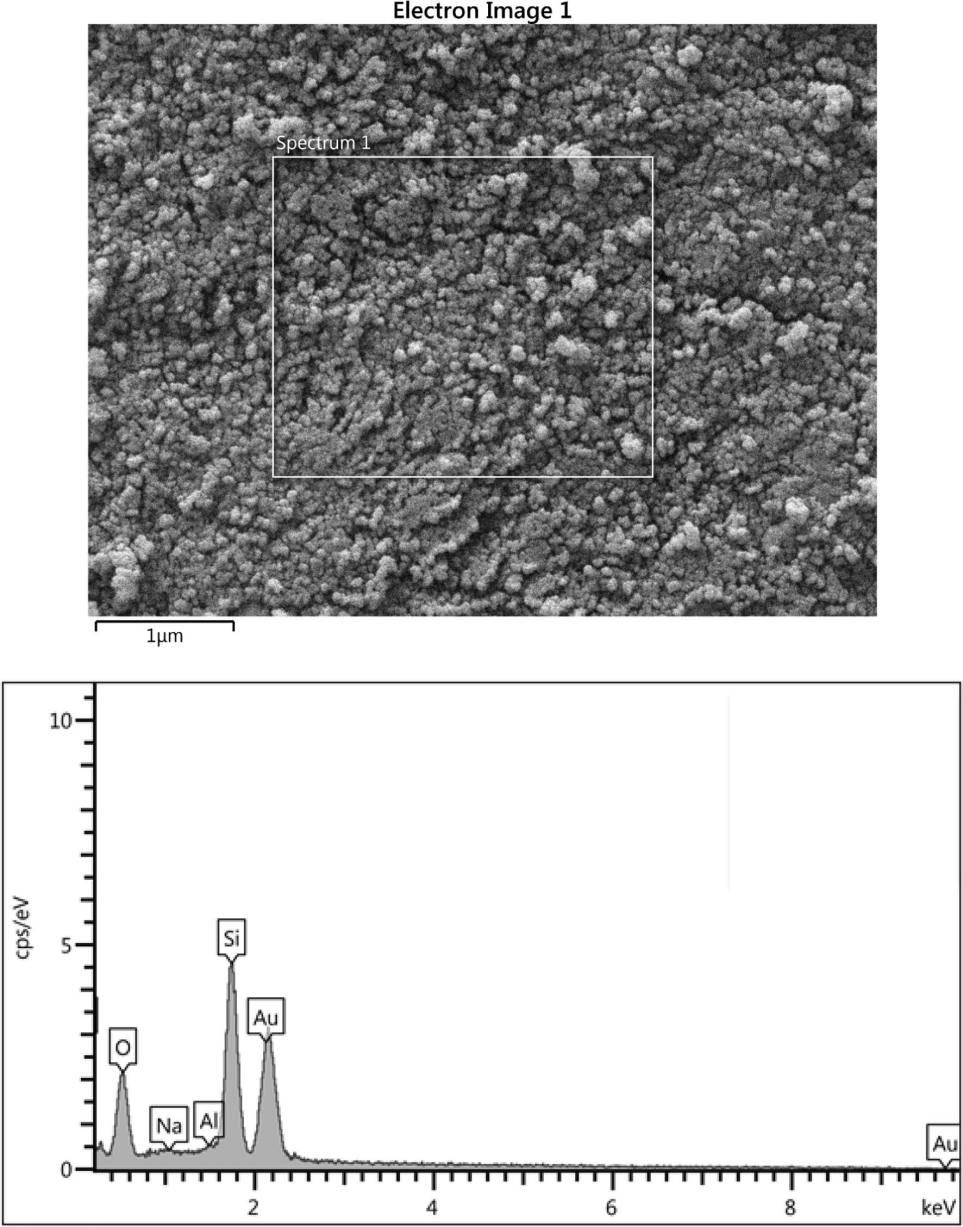
SEM scans and EDS analysis of Al_2_O_3_-MSU-S.

SEM image and EDS of the sample are illustrated in Fig. 5. The Al_2_O_3_-MSU-S sample shows aggregates of rounded particle^3^. The EDS results in the images verifies successfully loading of Al_2_O_3_ on the surface of adsorbent.

### Adsorption mechanism

As mentioned in other studies and research, sulfur compounds can be removed by certain sorbents either through the S–M bonds formation or the π-complexation. Direct sulfur-sorbent interactions by ion exchange between metal oxide (Al^3+^) and sulfur compounds can be attributed to hard and soft acids and bases (HSAB) principle. According to this theory, hard acids prefer to connect with hard bases, and soft acids prefer soft bases. The large differences in electronegativity between hard acids and hard bases leads to strong ionic interactions. The electronegativities of soft acids and soft bases are almost the same and therefore, they have less ionic interactions and they almost make covalent connections. The interactions between hard acid–soft base or soft acid–hard base are mostly polar covalent and tend to be more reactive or less stable. The polar covalent compounds readily make either ionic or covalent compounds if they are permitted to react. Al^3+^ is a hard acid and prefers to combine with DBT by the direct S–M (metal oxide) σ-bond. In this case, the strength of the direct S–M σ-bond basically depends on the charge number and ionic radius of the metal ion^21^.

### Adsorption equilibrium

Synthetic fuel with various amounts of DBT was applied to investigate the adsorption isotherms. The Freundlich, Temkin and Langmuir isotherms were applied to examine equilibrium results. Equations (1)–(3) show the mathematical form of the isotherms:

1 $$ {\text{Langmuir isotherm}}: \,\,\,q_{e} = \frac{{q_{m} K_{L} C_{e} }}{{1 + K_{L} C_{e} }} $$

2 $$ {\text{Freundlich isotherm}}: q_{e} = K_{F } C_{e}^{\frac{1}{n}} $$

3 $$ {\text{Temkin isotherm}}:\,\,\, q_{e} = \frac{RT}{{b_{T} }}\ln A_{T } C_{e} $$

*C*_*e*_ (mg kg^−1^) is the content of sulfur-component material at equilibrium; *K*_*L*_ (kg mg^−1^) and *q*_*m*_ (mg g^−1^) are the Langmuir isotherm parameters relevant to the adsorption energy and the largest load, respectively; *K*_*F*_ (mg^1−(1/n)^/g^−1^ kg^1/n^) and *1/n* show the Freundlich parameters relevant to the adsorption volume and also in Temkin ; *R* is universal constant, *T* is temperature, $A_{T }$(L/g), and $b_{T}$ are Temkin constants, respectively; and *q*_*e*_ (mg g^−1^) is the content of sulfur-component adsorbed at the equilibrium/mass of adsorbent. Equations (4)–(6) show the linearized form of the Langmuir, Freundlich and Temkin models.

4 $$ {\text{Langmuir isotherm}}:\,\,\frac{1}{{q_{e} }} = \frac{1}{{q_{m} }} + \left( {\frac{1}{{K_{L} q_{m} }}} \right)\frac{1}{{C_{e} }} $$

5 $$ {\text{Freundlich isotherm}}:\,\,\,\,lnq_{e} = lnK_{F} + \frac{1}{n}lnC_{e} $$

6 $$ {\text{Temkin isotherm}}: q_{e} = \frac{RT}{{b_{T} }}\ln A_{T } + \frac{RT}{{b_{T} }}\ln C_{e} $$

**Figure 6:**
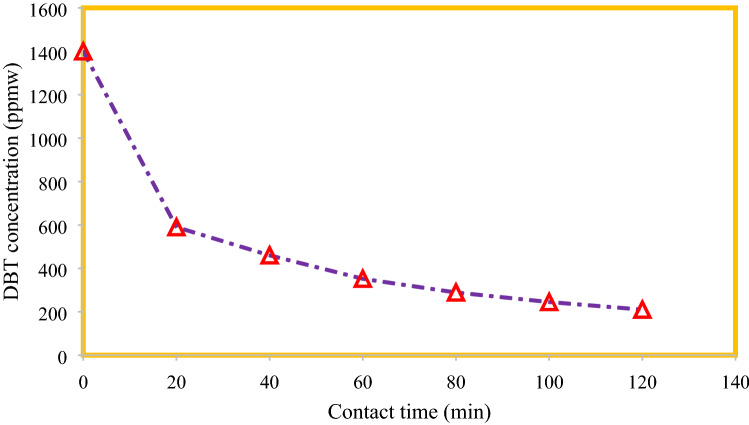
Contact time effect on Al_2_O_3_-MSU-S execution.

**Figure 7:**
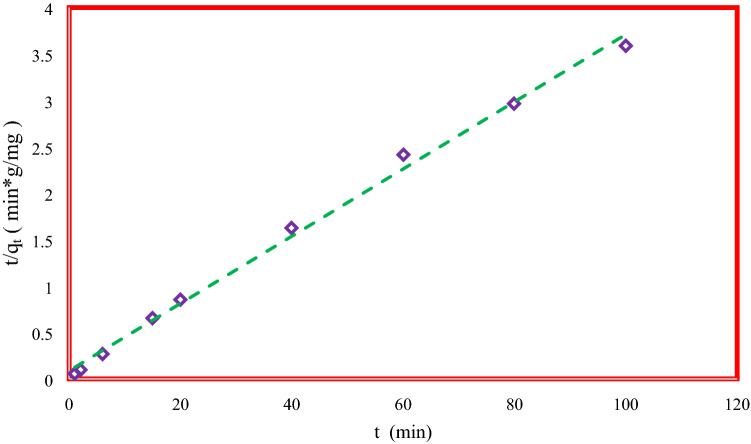
Pseudo–second order performance to predict experimental data.

**Figure 8:**
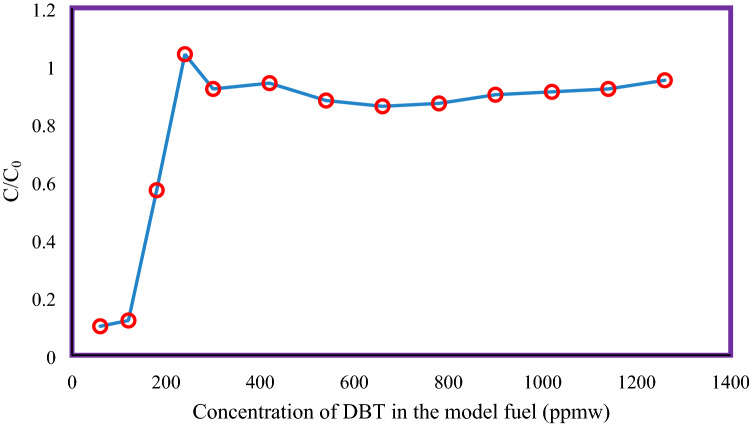
Breakthrough curves of adsorption of DBT on Al_2_O_3_-MSU-S (1 atm, 25 °C, 1.0186 h^−1^).

*K*_*L*_ and *q*_*m*_ for Eq. (4), also *K*_*F*_ and 1/n in Eq. (5), $$ \frac{RT}{{b_{T} }}$$ and $$A_{T } $$ for Eq. (6) can be found using slope and intercept of the plotted experimental data. Figures 6, 7, 8 illustrate the linear form of models fitted on the experimental data^2,19^.

Tables 2 and 3 demonstrates the calculated parameters from regression of data and with the correspondence coefficient (*R*^*2*^) amounts of the models for adsorption isotherms for MSU-S and Al_2_O_3_-MSU-S. It is obvious that Temkin model can be used to define the adsorption of DBT on the sorbent due to higher values of *R*^*2*^ compared with other models. Based on this model, there is an indirect adsorption interaction between DBT and Al_2_O_3_-MSU-S as adsorbent. It means the heat of adsorption decreases with increasing coverage in a linear relation. Therefore, a uniform distribution of binding energies was observed in the adsorption process.

**Table 2:** Different isotherm models for DBT adsorption on MSU-S.

Isotherm model	Parameters	Compound (DBT)
Langmuir	q_m_ (mg/g)	19.23
	K_L_ (kg/mg)	0.00226
	R^2^	0.9955
Freundlich	1/n	0.472
	K_F_ (mg/g) (kg/g)^1/n^	1.25
	R^2^	0.9732
Temkin	A_T_ (kg/g)	0.0184
	RT/b_T_	4.61
	R^2^	0.9963

**Table 3:** Different isotherm models for DBT adsorption on Al_2_O_3_-MSU-S.

Isotherm model	Parameters	Compound (DBT)
Langmuir	q_m_ (mg/g)	66.67
	K_L_ (kg/mg)	0.00106
	R^2^	0.8450
Freundlich	1/n	0.437
	K_F_ (mg/g) (kg/g)^1/n^	1.65
	R^2^	0.8990
Temkin	A_T_ (kg/g)	0.013
	RT/b_T_	13.057
	R^2^	0.9100

### Kinetics of DBT adsorption

From Fig. 6 it is clear that by increasing the contact time between model fuel and adsorbent, the residual concentration of sulfur content decreased. This figure points out that the major and effective adsorption takes place in the first 40 min and after a while reaches to an asymptotic amount.

Pseudo-first order, pseudo-second order, and intraparticle diffusion kinetic relations have been utilized to assess the kinetics of sulfur adsorption onto Al_2_O_3_-MSU-S.

The pseudo-first order relation is shown as:

7 $$ \frac{{dq_{t} }}{dt} = k_{lad} \left( {q_{e} - q_{t} } \right) $$

*q*_*t*_ = adsorption loads (mg/g) at the time of *t*, *q*_*e*_ = adsorption loads (mg/g) at equilibrium, *k*_*1ad*_ = constant rate of pseudo-first order adsorption (per minute).

Integrating from Eq. (7) and employing boundary limits from *t* = 0 to *t* = *t* and *q*_*t*_ = 0 to *q*_*t*_ = *q*_*t*_, leads to the following equation:

8 $$ ln\left( {q_{e} - q_{t} } \right) = lnq_{e} - k_{lad} t $$

As shown in Eq. (8) if *ln (q*_*e*_* − q*_*t*_*)* vs. time is plotted, *k*_*1ad*_ and *lnq*_*e*_ could be defined through slope and intercept of the model, respectively. Equation (9) shows the pseudo-second-order kinetic reltion^34–36^:

9 $$ \frac{{dq_{t} }}{dt} = k_{2ad} \left( {q_{e} - q_{t} } \right)^{2} $$

where *k*_*2ad*_ expresses the rate constant (gr/mg.min). After being integrated and employing boundary values (*t* = 0 to *t* = *t* and *q*_*t*_ = 0 to *q*_*t*_ = *q*_*t*_), equation can be derived in the following^35–38^:

10 $$ \frac{1}{{q_{t} }} = \frac{1}{{k_{2ad} q_{e}^{2} }} \times \frac{1}{t} + \frac{1}{{q_{e} }} $$

As shown in Eq. (10) if *t/q*_*t*_ vs. *t* is plotted, *k*_*2ad*_ and *q*_*e*_ are defined through slope and intercept of the model. The intraparticle pattern which was presented by Weber and Morris is indicated as follows^39,40^:

11 $$ q_{t} = k_{i} t^{0.5} + C $$

where *k*_*i*_ shows the rate constant (mg/g min^0.5^) and *C* is a constant (mg/g). As shown in Eq. (11) if *q*_*t*_ vs. *t*^0.5^ is plotted, *k*_*i*_ and *C* can be specified through slope and intercept of the model, respectively. Tables 4 and 5 illustrates the constant parameters and *R*^*2*^ values of models which are calculated by curve fitting of experimental data for MSU-S and Al_2_O_3_-MSU-S. As it is observed clearly, the best fitting for adsorption experimental data is the pseudo-second order pattern with maximum correlation coefficients in comparison with the intraparticle diffusion pattern and pseudo-first order models^18,39,40^. Figure 7 shows the performance of pseudo second order model to predict experimental data. Regarding the experimental and theoretical study, *k*_*2*_, the pseudo-second order constant is related to the primary concentration of the adsorbate. From this model, it can be suggested that the adsorption procedure is in two-steps. The first stage exhibits the film diffusion from mixture to the outer surface of adsorbent and the second step indicates diffusion into the rough surface and abundant voids. One of the major advantages of this model is capability of modeling adsorption with significant change in adsorbate concentration which makes it reliable.

**Table 4:** Kinetical study for adsorption of DBT on MSU-S.

Compounds	Pseudo first-order			Pseudo second-order			Intraparticle diffusion		
	q_e_ (mg/g)	k_1ad_ (1/min)	R^2^	q_e_ (mg/g)	k_2ad_ (g/mg.min)	R^2^	q_e_ (mg/g)	k_i_ (g/mg.min^0.5^)	R^2^
DBT	15.16	0.032	0.9659	15.92	0.00225	0.9813	1.453	1.026	0.8765

**Table 5:** Kinetical study for adsorption of DBT on Al_2_O_3_-MSU-S.

Compounds	Pseudo first-order			Pseudo second-order			Intraparticle diffusion		
	q_e_ (mg/g)	k_1ad_ (1/min)	R^2^	q_e_ (mg/g)	k_2ad_ (g/mg.min)	R^2^	q_e_ (mg/g)	k_i_ (g/mg.min^0.5^)	R^2^
DBT	9.99	0.011	0.9095	11.40	0.213	0.9958	19.10	0.8681	0.9031

### Fixed-bed adsorption/breakthrough test

#### *Adsorption of DBT over Al*_*2*_*O*_*3*_*-MSU-S*

Figure 8 shows the breakthrough graphs of adsorption of DBT on Al_2_O_3_-MSU-S at ambient temperature, atmospheric pressure and 1.0186 per hour LHSV. For these experiments 2.92 g of the model fuel/gram of sorbent (g-F/g-A) was used in the fixed-bed adsorber. When saturation point is reached, the *C/C*_*o*_ value for DBT rises strongly.

In order to find saturation capacity of Al_2_O_3_-MSU-S, integral calculus of the breakthrough curve is used. It was observed that adsorption load of presented sorbent to adsorb DBT from model fuel is 0.072 mmol/g^7^. Table 6 shows the adsorption capacity of different loads of metals on MSU-S adsorbent. It is obvious that the maximum adsorption load of DBT is related to Al_2_O_3_-MSU-S adsorbent.

**Table 6:** Adsorption capacity for DBT on different adsorbent.

Adsorbent	Adsorption capacities (mmol/g)
MSU-S^6^	0.034
CrO_2_-MSU-S^6^	0.068
Fe_2_O_3_-MSU-S^6^	0.047
CeO_2_-MSU-S^21^	0.013
Cu_2_O-MSU-S^21^	0.038
Al_2_O_3_-MSU-S (present work)	0.072

## Conclusion

In this work, MSU-S was synthesized, modified with aluminum and characterized to be applied in adsorptive desulfurization of model fuel. Modification of present MSU-S by the aluminum had negative effect which decreased the surface area for adsorption. Loading aluminum ions on the MSU-S enhanced adsorption selectivity through ion-exchange in the adsorbent. Comparing between aluminum modification and other metal modified MSU-S showed that capacity drastically varied by the order of Al_2_O_3_ > CrO_2_ > Fe_2_O_3_ > Cu_2_O > CeO_2_. Finally, it was found that Temkin and pseudo-second order models are the best models to define DBT adsorption on Al_2_O_3_-MSU-S equilibrium and kinetics phenomena.
